# Unintentional Injuries Are Associated with Self-Reported Child Maltreatment among Swedish Adolescents

**DOI:** 10.3390/ijerph20075263

**Published:** 2023-03-25

**Authors:** Ylva Tindberg, Staffan Janson, Carolina Jernbro

**Affiliations:** 1 Department of Women’s and Children’s Health, Uppsala University, 751 85 Uppsala, Sweden; 2 Centre for Clinical Research Sörmland, Uppsala University, 631 88 Eskilstuna, Sweden; 3 Division of Public Health Sciences, Department of Health Sciences, Karlstad University, 651 88 Karlstad, Sweden

**Keywords:** child maltreatment, child abuse, poly-victimization, unintentional injuries, healthcare, dental care, adolescents

## Abstract

Injuries constitute a large share of childhood morbidity and mortality. This study examines whether adolescents with self-reported experiences of different types of child maltreatment more frequently reported unintentional injury events requiring health- or dental care during the last year and/or hospitalization at any time during childhood. Cross-sectional data from a Swedish national representative school survey (2016) including 4741 adolescents were used (78.5% response rate). Data were analyzed with univariate tests and multiple logistic regression. Statistically significant associations between reported experiences of physical, psychological and sexual abuse, neglect, and witnessing partner violence during childhood and reported unintentional injuries requiring health- or dental care during the last year were found (aOR:s between 1.39–1.77). The corresponding association for poly-victimization was aOR 1.91 (95% CI 1.39–2.62). Furthermore, a linear-by-linear association was seen for degree of victimization and number of episodes of unintentional injuries that required care in the last year (*p* = 0.000), as well as lifetime hospitalizations (*p* = 0.000). This study shows significant associations between child maltreatment and unintentional injuries requiring health- and/or dental care and hospitalization. To improve both injury and child maltreatment prevention, healthcare professionals need to pay particular attention to children and adolescents who repeatedly seek healthcare services due to injurious events.

## 1. Introduction

Injuries constitute a large share of childhood and adolescent morbidity and mortality in the European Region, with increasing incidence by age [1,2]. In preschool children, most injuries occur in traffic or at home involving falls, poisonings and burns [2,3], while injuries among adolescents involve falls in sports, peer violence, motor vehicles, self-inflicted injuries and suicides [1,2,3,4]. Many of these outcomes are in adolescence related to risk-taking, use of alcohol or drugs or both [1,2]. In the UK, over half of the deaths among 15-18-year-olds can be attributed to external causes together with risks and behaviors, whereas adolescents with chronic conditions, including mental and behavioral disorders, account for more than a third [2].

In adolescence, experimenting and taking risks is natural part of the psycho-social development required for the transition from child- to adulthood, but may in some cases develop to health-compromising behavior [5]. Chronic conditions, especially neuropsychiatric variations, such as ADHD, including impaired impulse control, impaired perception, and thus, impaired perception of danger, are related to risk-taking to a high degree [5,6]. Another vulnerable group of adolescents with a high degree of risk-taking are those having experienced child maltreatment [7,8].

Adverse childhood experiences, and especially child maltreatment, have been linked to behavior problems, health-compromising behavior and adverse health outcomes [7,8,9,10,11]. Studies have also shown a substantial overlap between different forms of child maltreatment [7,11]. Furthermore, it is known that the degree of exposure to several types of maltreatment, i.e., poly-victimization, in childhood is associated to the degree of lower quality of life, poorer health and more health-compromising behavior in young people [7,8,11].

Lower socioeconomic status [10,12,13], living with a single parent [13,14], parental drug and alcohol problems [10], having been the subject of a report to child protective services [15], bullying [16,17] and child- and adolescent attributes such as hyperactivity [18,19] are all factors known to be associated with child maltreatment and unintentional injuries in children. Nevertheless, scant research has focused on the association between child maltreatment and unintentional injuries, although previous national surveys among Swedish school children have highlighted the issue [20]. The known relationship between child maltreatment and health-compromising behaviors, on the one hand, and health-compromising risk-taking and unintentional injuries, on the other hand [21], urges exploration of a possible connection between child abuse and unintentional injurious events.

The aims of the present study were to examine (1) the association between different types of self-reported child maltreatment, and unintentional injuries requiring health- or dental care during the last year; (2) the association between child maltreatment, including poly-victimization, and hospitalization during life; and (3) if there is a possible dose-response-relation between child maltreatment and unintentional injuries. For this, we used the Swedish definition of child maltreatment by The Committee Against Child Abuse [20], which includes when an adult subjects a child to physical or psychological violence, including witnessing intimate partner violence, sexual abuse or neglect.

## 2. Materials and Methods

### 2.1. Data Collection and Study Population

The current study was based on cross-sectional data from a Swedish national survey on child maltreatment performed in 2016 and including 4741 adolescents [20]. The methodology and ethics were described in an earlier publication [22].

The data was anonymously collected in schools, in primary school Grade 9 (15-year-olds) and in the second year of high school (17-year-olds), in the autumn of 2016. A sampling frame, consisting of 1561 primary schools and 1320 high schools, was created based on the Swedish National school register. To ensure that schools of different sizes were included in the sampling, the schools were stratified based on the number of students [22].

The final sample consisted of 313 primary schools and 440 high schools. Of these, 142 schools participated (71 schools per grade), representing a participation rate of 22.7% of the sampled primary schools and 16.1% of the high schools. By far the most common reason for school non-participation was the lack of time of teachers and principals. A few schools declined participation because of the risk of upsetting students by the content of the survey. Altogether, 273 classes participated in the study. The total response rate was 78.5%; 82.2% in primary schools and 75.5% in high schools. 

### 2.2. Measures

The main independent variables used in the current study are described in more detail in Supplementary Table S1. *Physical and psychological abuse* were measured with questions based on the validated instrument Conflict Tactics Scale, Parent and Child version [23], as used in several previous Swedish child maltreatment surveys. *Sexual abuse* (by an adult perpetrator) was assessed using questions based on instruments from a previous Swedish study on child sexual abuse [24]. *Neglect* was assessed by an instrument with ten questions about emotional and physical neglect used in the ACE study [25]. *Witnessing partner violence*, both psychological and/or physical, was measured with a question that had been used in a Swedish population-based study [26]. *Any child maltreatment* was considered if any of the above was deemed present, being in line with Swedish definition and Swedish law. *Poly-victimization* was defined as being exposed to three to five forms of child maltreatment measured in this study. 

Covariates used in adjusted analyses were age, gender, birth region, family structure and disability. Any *disability* was considered if a diagnosis of either visual impairment, hearing impairment, epilepsy, mobility impairment, attention deficit hyperactivity disorder (ADHD) or autism was reported. Further description of the variables *family structure* and *family economy* are found in Supplementary Table 1.

### 2.3. Injuries and Accidents

To measure the prevalence of unintentional injuries requiring health- or dental care during the last year, the following question was used: “*During the last year, have you been injured in an accident in such a way that you had to visit your GP/family doctor, dentist, or hospital?*”. The response alternatives were “No”, “Yes, once” and “Yes, many times”. 

To measure the prevalence of unintentional injuries requiring hospitalization (hospital stay) during childhood, the following question was used: “*Have you ever been injured in an accident in such a way that you needed hospitalization?*”. The response alternatives were “No”, “Yes, once”, “Yes, many times” and “Don’t know”.

Intentional injuries, i.e., injuries due to violence, were measured by the following question: *Have you ever been injured due to violence from any person that required health- or dental care?* The response alternatives were “No”, “Yes, once” and “Yes, many times”.

### 2.4. Analysis

Data were analyzed with univariate tests (Chi-2), and with binomial logistic regression models, expressed as crude odds ratio (OR) and adjusted odds ratio (aOR) with 95% confidence intervals (CI). *p*-values < 0.05 in two-tailed analysis were considered as statistically significant. The analyses were performed in IBM SPSS Statistics 22.

### 2.5. Ethical Considerations

Study participants received information beforehand about the study’s purpose, that participation was voluntary and the right to stop participating at any time. Parents of Grade 9 students also received this information and were given the opportunity to disallow their child to participate (if the child was under the age of 15). There was only one case in which a parent hindered his/her child to participate. Participants provided informed consent by answering the questionnaire. Information was given where participants could receive counselling if needed and the preparedness among the school health staff was increased at the time of the survey. This study was approved by the Regional Research Ethics Committee at Stockholm University (reg. no 2016/1014-31).

## 3. Results

The study population is described in Table 1. Gender and age groups were equally represented. One in five students were living with a single parent and one in ten were born outside of the Nordic region. Poor family economy was reported by 3.3%, and 14.8% children reported a disability. 

### 3.1. Unintentional Injuries Requiring Health- or Dental Care

Of the total sample, 21.5% adolescents reported suffering any unintentional injury that had led to health- or dental care during the last year (Table 2). Reported injurious events had predominantly occurred in sports during leisure time (7.9%), at home (4.1%) and at sports during school time (2.5%). In total, 5.6% had repeatedly suffered unintentional injuries requiring health- or dental care during the last year. Furthermore, 25.8% of the respondents reported hospitalization due to unintentional injuries at some point in life (Table 2). Repeated hospitalization due to unintentional injuries was reported by 5.8% of adolescents. Furthermore, 6.8% of the adolescents reported intentional injuries from violence requiring health- or dental care at some point in life, the majority due to peer violence (data not shown).

As shown in Table 2, younger age, living with a single parent, being born outside of the Nordic region, poor family economy and reporting disability were significantly higher associated with unintentional injuries requiring health- or dental care in the last year. Being a boy and reporting disability were significantly associated with an increased need for hospitalization due to unintentional injuries during life.

### 3.2. Child Abuse

In total, 24.4% of the adolescents reported physical abuse (CPA) by any adult during childhood. Severe CPA, such as having been severely or frequently beaten, was reported by 11.5%. Psychological abuse at some point during childhood was reported by 15.7% and emotional or physical neglect by 6.2%. Sexual abuse by any adult was reported by 8.5%. Witnessing partner violence (WPV) was reported by 14.2% of the entire group. In total, 43.7% had been exposed to any type of maltreatment during their lifetime, whereas 22.3% had been exposed to three or more types of maltreatment (poly-victimization), corresponding to 8.4% of the total sample.

As seen in Table 2, being a girl, older age, living with a single parent, being born outside of the Nordic region, poor family economy and reporting disability were all associated to experiences of exposure to any kind of child maltreatment during childhood.

### 3.3. Child Abuse and Unintentional Injuries Requiring Different Types of Care

Adolescents who reported any child maltreatment during childhood were significantly more likely to report unintentional injuries requiring health- or dental care in the last year than peers who were not exposed (Table 3). The analyses remained significant after adjustments for gender, grade, family structure, birth region, family economy and disability. 

A need for health- or dental care due to an unintentional injury in the last year was significantly more common among adolescents with experiences of child physical abuse (aOR 1.77), psychological abuse (aOR 1.64), sexual abuse (aOR 1.66), witnessing intimate partner violence (aOR 1.39) and parental neglect (aOR 1.51) than among peers not sharing these experiences (Table 3). In total, for adolescents having reported any type of child maltreatment during childhood, the aOR was 1.67 (95% CI 1.41–1.99) for injurious events leading to health- or dental care in the last year as compared to peers not reporting child maltreatment. 

There was a significant association between intentional injuries and unintentional injuries (data not shown). Among the adolescents reporting injuries from violence (i.e., intentional injuries) requiring health- or dental care; 47.4% had also required health care for unintentional injuries in the last year compared to 19.1% of those who had no intentional injuries (*p* < 0.001). 

Adolescents that had experienced poly-victimization were twice as likely to have visited healthcare facilities or a dentist due to an uninflected injurious event in the last year, with an aOR of 1.91 (95% CI 1.39–2.62) (Table 3) compared to peers without these experiences. 

Adolescents that had reported any type of child maltreatment also more frequently reported unintentional injuries requiring hospitalization at some point during life with an aOR of 1.48 (95% CI 1.25–1.75) compared to peers that had not been exposed. For poly-victimization, the corresponding aOR was 1.96 (1.41–2.73), and for neglect it was 2.16 (1.53–3.04). Hospitalization due to unintentional injuries during life had a border-line association to witnessing intimate partner violence, while there was no statistical association to sexual abuse (Table 4).

In Table 5, a dose-response relationship between child maltreatment and injuries is shown. The more frequent the exposure to different types of maltreatment and abuse, the more often injuries leading to health- or dental care in the last year and lifetime injuries leading to hospitalization.

## 4. Discussion

The current nationally representative study of Swedish 15–17-year-old adolescents found consistent associations between all types of child maltreatment and unintentional injuries leading to health- or dental care in the last year and to hospitalization at some point during life. We also noted a dose-response relationship between the degree of exposure to several types of child maltreatment and unintentional injurious events. 

To the best of our efforts, we searched the literature for research on a relationship between child maltreatment and unintentional injuries, but found little. Studies in the clinical setting have pointed at the influence of parenting behavior and supervisory neglect [27,28], which is in line with our findings of doubled odds for unintentional injuries leading to health- or dental care in the last year among adolescents reporting parental neglect. 

The injury pattern for adolescents, as compared to younger school children, is known to change according to a more experimental and sometimes risk-taking behavior that might include motor vehicles, alcohol, drugs and interpersonal violence [1,3,4,12]. Part of this pattern is explained by adolescent’s natural development involving experimenting and exploration, for own future references and to become accepted among peers; however, sometimes developing into a health-compromising behavior [5]. ADHD and its covariation of inattention, hyperactivity and impulsivity, is a complicating condition in adolescence, resulting in increased risk-taking as compared to counterparts without these problems [6]. Furthermore, a relevant pathway is the heredity of neuropsychiatric variations such as ADHD [29], for instance, poor impulse control, which may be a risk factor for parental violent behavior as well as injury proneness in children and adolescents. Child maltreatment is another aggravating circumstance, not only causing immediate physical and psychological damage, but also affecting the child’s future life by its association to risk-taking and health-compromising behaviors [7,8,10]. Furthermore, chronic conditions, including ADHD and mental disorders, have been associated with an increased risk of child maltreatment [18]. Our present findings are supported by the well-established relation between child maltreatment and health-compromising behavior, such as usage of alcohol or drugs, most likely increasing the odds for unintentional injuries, contributing to the poorer health outcome for this vulnerable group. [7,8,9,10]. 

As pointed out in previous research, the strong association between child maltreatment and poorer health cannot be explained by a single cause, since health outcomes are complex and depend on multiple factors [7,30]. The present study focuses on the association between child maltreatment and unintentional injuries, but the theoretical framework and the pathways described by Kendall-Tackett can also be applied here [30]. The described behavioral pathway includes various types of health-compromising behaviors, such as substance abuse and sleeping difficulties, affecting the individual’s risk-taking, lack of judgement and reactivity. Furthermore, the cognitive pathway refers to the internal mental framework by which a person interprets stressful life events, as well as the motives and actions of others. The internal working model also affects a person’s beliefs regarding how much power they have in situations and how much they can do to help themselves. Finally, the emotional pathway includes depression, with or without PTSD, and lack of sleep, all negatively affecting self-worth and increasing the risk of injury or death because of accidents at home, at work or due to traffic [30].

The strong association between unintentional and intentional injuries requiring health care is an interesting finding. Being victimized by peers is known to have effects on mental health [31], but is also believed to work as a short-term injury trigger in these settings. Disturbed concentration and attention are suggested to result in peripheral narrowing and slower vision reaction time during stress [16]. A child with experiences of maltreatment at home, or elsewhere, are likely to be triggered by the same mechanisms. 

Another finding strengthening our result are the strong associations between poly-victimization reported by adolescents and having experienced unintentional injurious events in need of healthcare or seeing a dentist in the last year and requiring hospitalization at some point during life. These findings were supported by statistically significant linear-by-linear relationships between the degree of victimization and unintentional injuries. These associations point at the need for healthcare professionals to pay particular attention to children and adolescents seeking health- or dental care and ending up at hospital due to unintentional injurious events, especially those who repeatedly seek care.

The United Nations Convention on the Rights of the Child declares that children and adolescents need special consideration to safeguard their right to health and to a safe environment, free from injury and violence. The WHO further states that no violence against children is justifiable and all forms of violence are preventable [32]. The same position applies to accidents and injuries, which can also be prevented in many cases [1]. The present findings of strong associations between child physical abuse, psychological and sexual abuse, witnessing intimate partner violence, and parental physical and emotional neglect and unintentional injury prevalence in children and adolescence call for continued efforts to address both primary and secondary prevention. To obtain a deeper understanding of the pathways from various types of child maltreatment to unintentional injuries, more research is warranted. This would be helpful in the prevention of unintentional injuries, but it would also highlight the need for reinforced child maltreatment prevention. 

### Strengths and Limitations

The present study has some strengths and limitations. The strengths of the present study include the large, nationally representative sample and the high response rate of 79%. Furthermore, the internal dropouts for important key questions regarding the different types of maltreatment, except from sexual abuse, and injuries needing care were generally low.

Another strength of our study is the use of the Swedish definition of child maltreatment, including IPV [20]. The association between IPV and poorer health in childhood and later in life is likely to support our findings of an association between child maltreatment and unintentional injuries [20]. A limitation is that the results may be more difficult to compare with studies from other countries where IPV is differently viewed, both legally and in research.

Limitations of the present study include its concentration on unintentional injurious events over the preceding year. Furthermore, the results do not tell whether child maltreatment and hospitalization due to unintentional injuries occurred early in life or among school children. One thought, however, is that the relationship found between child maltreatment and unintentional injuries requiring health care in the last year indicate that maltreatment in any form was an ongoing part of the young respondent’s everyday life. Our noted association between required health care for unintentional injuries among adolescents reporting need for health care due to intentional injuries (*p* < 0.001) would support this. 

Furthermore, the study does not provide information about injury type. This calls for further research to examine different types of injuries. Still, the associations found between child maltreatment and unintentional injuries are both strong and show a dose-response relationship.

Data collected in 2016 may not reflect the situation of today. Changes in the prevalence of either child maltreatment or unintentional injuries, for instance, influenced by the COVID-19 pandemic, could have changed, but might not have affected the associations. In our previous national study on child maltreatment conducted in 2011, we saw the same pattern of associations as in this study from 2016.

Maltreatment and harassment in any form may be sensitive to deal with. Consequently, there is a risk of recall bias, which may result in the underreporting of maltreatment. This would be expected to lead to an underestimation of the associations. We used a questionnaire based on the Conflict Tactics Scale, which approaches abusive behaviors in a graded manner starting with less serious maltreatment moving on to more severe forms of abuse [23]. 

## 5. Conclusions

In conclusion, injuries constitute a large share of adolescent morbidity and mortality worldwide. This school-based study shows that unintentional injurious events among 15–17-year-olds requiring health- or dental care in the last year was associated with child physical abuse, psychological and sexual abuse, witnessing intimate partner violence, neglect and, particularly, with poly-victimization during childhood. We also noted a strong and dose-response relationship between the degree of victimization and unintentional injuries. The present study adds to the knowledge regarding the association between child maltreatment and unintentional injuries, and is of importance to several stakeholders, such as professionals in healthcare, social services and schools. To improve both injury and child maltreatment prevention, healthcare professionals need to pay particular attention to children and adolescents seeking health-or dental care due to unintentional injurious events, especially those who repeatedly seek care.

## Figures and Tables

**Table 1:** Socio-demographic characteristics of sample.

Sample Characteristics	n	%
**Gender**		
Boy	2331	50.7
Girl	2270	49.3
**Grade**		
Grade 9 in primary school (15 y)	2264	48.4
Year 2 in high school (17 y)	2417	51.6
**Family structure**		
Live with both parents who live together	2966	65.5
Parents separated—alternate living arrangements	498	11.0
Live mostly or exclusively with one parent	995	21.9
Foster care	72	1.6
**Birth region**		
Nordic countries	4184	88.7
Outside the Nordic region	532	11.3
**Family economy**		
Good economy	4528	96.7
Poor economy	153	3.3
**Disability**		
No	3748	85.2
Yes	649	14.8

**Table 2:** Sociodemographic variables and the association with any child maltreatment during life, to unintentional injuries requiring health- or dental care during the last year and to unintentional injuries requiring hospitalization at some point during life.

Sample Characteristics	Exposure to Any Child Abuse during Life % (n)	Injury Requiring Health- or Dental Care during the Last Year % (n)	Injury Requiring Hospitalization during Life % (n)
**Gender**			
Boy	41.2 (798)	22.7 (522)	31.6 (680)
Girl	44.2 (903) *	20.1 (450)	19.6 (412) ***
**Grade**			
Grade 9 in primary school (15 y)	39.0 (750)	23.3 (518)	24.8 (513)
Year 2 in high school (17 y)	47.6 (1009) ***	19.8 (475) **	26.7 (602)
**Family structure**			
Living with both parents	37.2 (1110)	20.1 (691)	25.0 (809)
Single/other (i.e., foster care)	61.4 (579) ***	24.0 (250) **	27.2 (262)
**Birth region**			
Nordic countries	41.3 (1502)	20.7 (859)	25.8 (999)
Outside the Nordic region	62.6 (273) ***	27.9 (142) ***	25.4 (122)
**Family economy**			
Good economy	42.1 (1650)	20.8 (932)	25.7 (1077)
Poor economy	83.3 (110) ***	39.6 (59) ***	31.4 (43)
**Disability**			
No	38.9 (1249)	19.9 (735)	23.2 (810)
Yes	58.7 (335) ***	27.2 (173) ***	35.6 (206) ***

Note: Statistically significant values as follows: * *p* < 0.05; ** *p* < 0.01; *** *p* < 0.001.

**Table 3:** The association between different types of child maltreatment (during life) and self-reported unintentional injuries leading to health- or dental care in the last year.

	Injuries Leading to Health- or Dental Care Last Year			
Types of Maltreatment		% (n)	OR (CI)	adjOR (CI) *
Any type of maltreatment	No	16.8 (386)	1.0	1.0
	Yes	27.2 (479)	1.85 (1.59–2.16)	1.67 (1.41–1.99)
Physical abuse	No	17.9 (595)	1.0	1.0
	Yes	30.1 (320)	1.97 (1.68–2.31)	1.77 (1.47–2.13)
Psychological abuse	No	19.1 (729)	1.0	1.0
	Yes	32.0 (224)	2.00 (1.66–2.37)	1.64 (1.32–2.05)
Sexual abuse	No	20.6 (802)	1.0	1.0
	Yes	29.2 (106)	1.59 (1.25–2.02)	1.66 (1.25–2.22)
Neglect	No	20.4 (823)	1.0	1.0
	Yes	31.9 (84)	1.83 (1.40–2.40)	1.51 (1.07–2.12)
Witnessing IPV	No	20.0 (774)	1.0	1.0
	Yes	28.4 (180)	1.59 (1.31–1.92)	1.39 (1.11–1.76)
Poly-victimization	No	19.2 (647)	1.0	1.0
	Yes	34.7 (107)	2.24 (1.75–2.88)	1.91 (1.39–2.62)

* Adjusted for adolescents’ gender, grade, family structure, birth region, family economy and disability.

**Table 4:** The association between different types of child maltreatment (during life) and self-reported unintentional injuries leading to hospitalization during life.

	Injuries Leading to Hospitalization during Life			
Types of Maltreatment		% (n)	OR (CI)	adjOR * (CI)
Any type of maltreatment	No	21.6 (472)	1.0	1.0
	Yes	30.7 (495)	1.60 (1.38–1.86)	1.48 (1.25–1.75)
Physical abuse	No	22.5 (705)	1.0	1.0
	Yes	34.6 (337)	1.82 (1.56–2.13	1.58 (1.31–1.89)
Psychological abuse	No	23.8 (853)	1.0	1.0
	Yes	37.1 (235)	1.88 (1.58–2.52)	1.74 (1.40–2.18)
Sexual abuse	No	25.1 (918)	1.0	1.0
	Yes	26.2 (86)	1.06 (0.82–1.37)	1.18 (0.88–1.62)
Neglect	No	24.5 (931)	1.0	1.0
	Yes	39.8 (92)	2.04 (1.55–2.52)	2.16 (1.53–3.04)
Witnessing IPV	No	24.9 (905)	1.0	1.0
	Yes	28.6 (163)	1.21 (1.0–1.74)	1.19 (0.94–1.52)
Poly-victimization	No	23.2 (737)	1.0	1.0
	Yes	36.9 (101)	1.93 (1.49–2.51)	1.96 (1.41–2.73)

* Adjusted for adolescents’ gender, grade, family structure, birth region, family economy and disability.

**Table 5:** Dose-response association between number of maltreatment types (during life) and unintentional injuries leading to health- or dental care in the last year and hospitalization during life.

Maltreatment	Unintentional Injuries Leading to Health- or Dental Care Last Year			Unintentional Injuries Leading to Hospitalization due to Injuries during Life		
	None % (n)	One % (n)	Many % (n)	None % (n)	One % (n)	Many % (n)
None	83.2 (1909)	13.6 (312)	3.2 (74)	78.4 (1712)	18.1 (395)	3.5 (77)
1–2 types	75.8 (816)	18.0 (194)	6.2 (67)	73.3 (729)	19.8 (197)	6.8 (68)
3–5 types	65.3 (201)	21.1 (65)	13.6 (42)	63.1 (173)	24.1 (66)	12.8 (35)

Linear-by-linear association *p* = 0.000.

## Data Availability

The dataset generated and analyzed in the current study is available from the authors, but not publicly available due to ethical guidelines.

## Supplementary Materials

The following supporting information can be downloaded at: https://www.mdpi.com/article/10.3390/ijerph20075263/s1, Supplementary Table S1—Description of main variables, including survey questions, items, response alternatives, recoding, and missing data.
