# Supplementary material for: Unintentional Injuries Are Associated with Self-Reported Child Maltreatment among Swedish Adolescents
Source: Int J Environ Res Public Health. 2023 Mar 25;20(7):5263. doi: 10.3390/ijerph20075263 (PMC10093922; doi:10.3390/ijerph20075263)
Supplement: Supplementary file 1 [file ijerph-20-05263-s001.zip › ijerph-2210124-supplementary.pdf]

Supplementary Table S1. Description of main variables, including survey questions, items, response alternatives, recoding and missing data.

Variables	Survey question and items	Response alternatives	Recoding	Missing data (%)
Physical abuse	Has any adult done any of the following to you and if so, how often: (1) Pulled by the hair or ear, (2) Smacked with an open hand, (3) Hit hard with an open hand or fist, (4) Kicked, (5) Burned or scalded (with hot liquid), (6) Grabbed by the throat/neck and choked, (7) Hit with a cane, belt, ruler or similar, (8) Threatened with a knife or firearm, (9) Injured with a knife or firearm.	(a) Never<br>(b) Few occasions<br>(c) Many times	***Any physical abuse:***<br>No: (a) on all items (1-9)<br>Yes: (b) or (c) on any item (items 1-9)<br>***Severe physical abuse:*** (b) or (c) on items 3-9<br>***Frequent physical abuse:*** (c) on any item	6.7
Psychological abuse	Has any adult done any of the following to you and if so, how often: (1) Insulted you (e.g. called you worthless, stupid, ugly) (2) Locked you in the basement, in a closet, or in a similar confined space, (3) Locked you out of the house, (4) Threatened to hit or hurt you (5) Treated you as if you didn't exist.	(a) Never<br>(b) Few occasions<br>(c) Many times	***Any psychological abuse:***<br>No: (a) on all items, or (b) on item 1 and/or 5<br>Yes: (b) or (c) on items 2-3 or (c) on items 1 or/and 5<br>***Severe psychological abuse:*** (b) or (c) on items 3-5	4.0
Sexual abuse	Has it happened that someone has done any of the following against your will? (1) Showed you images of you or other people in sexual positions, on the internet or via mobile phones, (2) Asked you to perform sexual acts on the internet, (3) Groped you or kissed you against your will, (4) Forced you to grope or kiss someone else, (5) Forced you to watch while someone else exposed his/her body or parts of his/her body to you, (6) Forced you to expose your body or parts of your body, (7) Forced you to have vaginal, anal, or oral sex.	(a) Never<br>(b) Few occasions<br>(c) Many times	No: (a) on all items<br>Yes: (b) or (c) on any item and responded that an adult was the perpetrator on the follow-up question about perpetrators.	9.4
Neglect	*Emotional neglect*: (1) Someone in my family helped me feel important or special, (2) I felt loved, (3) people in my family looked out for each other, (4) we felt close to each other, (5) my family was a source of strength and support, *Physical neglect:* (6) there was someone to take care of me, protect me, (7) take me to the doctor if I needed it, (8) I didn't have enough to eat, (9) my parents were too drunk or too high to take care of me, (10) I had to wear dirty clothes.	1= Never true<br>2= Rarely true<br>3= Sometimes true<br>4= Often true<br>5= Very often true	The responses were reversed-scored and summed, for emotional neglect (items 1-5) and physical neglect (items 6-10) respectively. For emotional neglect, respondents with scores of 15 or higher were considered neglected. For physical neglect, respondents with scores of 10 and higher were considered neglected. Any neglect was considered if the respondent had experienced emotional and/or physical neglect.	6.2
Witnessing Intimate Partner Violence	Has it occurred that you have seen or heard your father (or equivalent) do any of the following to your mother (or equivalent) during your childhood (1) used some form of physical violence (e.g. slapped her in the face, dragged her by the hair, threw things at your mother, struck with fist/a weapon, or kicked her), (2) Threatened your mother with physical violence, (3) Used words to insult, oppress, or dominate your mother? *The same question was repeated asking about the mother's violence towards the father*	(a) Never<br>(b) Few occasions<br>(c) Many times	No: (a) on items 1 and 2, (a) or (b) on item 3<br>Yes: (b) or (c) on items 1 and 2, or (c) on item 3	4.2

Injury last year leading to health- or dental care	During the last year, have you been injured in an accident in such a way that you had to visit your GP/family doctor, dentist, or hospital?	(a) Never<br>(b) Yes, once<br>(c) Yes, many times	In regression models, the responses were dichotomized:<br>No: (a)<br>Yes: (b) or (c)	1.4
Location at time of injury	If yes (on the question above), where did the accidents happen? School-yard, Physical Education (school); Other location at school; On the way to or from school; In town/center; At home; In the neighborhood; In traffic; Sports (leisure); At work			
Injury at any time leading to hospitalization	Have you ever been injured in an accident in such a way that you needed hospitalization?	(a) Never<br>(b) Yes, once<br>(c) Yes, many times<br>(d) Don't know	In regression models, the responses were dichotomized:<br>No: (a)<br>Yes: (b) or (c)<br>(d) was coded as missing	2.4
Family structure	Which adults do you live with? (1) Both my parents who live together; (2) Alternating living arrangements; (3) Living with mom mostly or solely; (4) Living with my mom and her new partner mostly or solely; (5) Living with my dad mostly or solely; (6) Living with my dad and his new partner mostly or solely; (7) Living in foster family; (8) other out of home care placement		Responses were dichotomized; living with both parents who live together or alternative living arrangements (item 1-2) versus all other responses (items 3-8)	4.4
Family economy	I feel that my family can afford to buy what we need	(a) Very true<br>(b) Somewhat true<br>(c) Somewhat untrue<br>(d) Very untrue	The responses were dichotomized:<br>Yes: (a) or (b)<br>No: (c) or (d)	1.3
